# Impact of cervical cancer on quality of life of women in Hubei, China

**DOI:** 10.1038/s41598-018-30506-6

**Published:** 2018-08-10

**Authors:** Niresh Thapa, Muna Maharjan, Yan Xiong, Daqiong Jiang, Thi-Phuong Nguyen, Marcia A. Petrini, Hongbing Cai

**Affiliations:** 1 Department of Gynecological Oncology, Zhongnan Hospital of Wuhan University, Hubei Cancer Clinical Study Center, Hubei Key Laboratory of Tumor Biological Behaviors, Wuhan, Hubei China; 2 Karnali Academy of Health Sciences, Jumla, Nepal; 3 Zhongnan Hospital of Wuhan University, HOPE School of Nursing, Wuhan, Hubei China; 4 Faculty of Nursing, Chiang Mai University, Chiang Mai, Thailand

## Abstract

We aimed to assess the quality of life (QOL) of the patients with cervical cancer after initial treatment, the factors affecting QOL and their clinical relevance. A total of 256 patients with cervical cancer who visited Zhongnan Hospital of Wuhan University from January 2017 to December 2017 were enrolled in this study. The European Organization for Research and Treatment of Cancer Quality of Life Questionnaire Core-30 item (EORTC QLQ-C30) and cervical cancer module (EORTC QLQ-CX24) was used to assess the QOL of patients. More than half of the patients with cervical cancer reported an excellent QOL. Symptoms mostly experienced were insomnia, constipation, financial difficulties, and menopausal symptoms. Global QOL and social functioning were statistically associated with education level, occupation, the area of living, family income and treatment modality. Similarly, role functioning showed significant association with the stage of cancer, treatment modality and time since diagnosis. The rural area of living and poor economic status of the patients with cervical cancer has a negative impact on overall quality of life. Younger and educated patients are more worried about sexuality. Patients treated with multiple therapies had more problems with their QOL scales than patients treated with surgery only.

## Introduction

Cervical Cancer (CC) is the fourth most common female cancer with estimated incidence and mortality of 528,000 and 266,000 respectively in the world^1^. China alone has around 18.7% (98,900 cases) of the total CC and 11.5% (30,500 deaths) of the total mortality in the world^2^. Although the overall incidence of CC in China is lower than African or South Asian countries, there are specific regions such as Hubei province, the central part of China, with one of the highest prevalence of CC^3^. Moreover, Wufeng county in Hubei province has the second highest incidence of CC and the highest mortality rate in China^4^.

Effective therapy for CC including surgery and concurrent chemoradiation can have a cure rate of 80% of women with early-stage disease [International Federation of Gynecology and Obstetrics (FIGO) stages I–II] and 60% of women with stage III disease^5^. The survival rate of CC in China is also increasing probably due to the free screening program that began in 2009. However, the quality of life after treatment has been primarily neglected^6^. The majority of patients with CC are diagnosed at a relatively younger age, and most of them have a long additional life expectancy with sequelae of the disease and its treatment. Therefore, the quality of life in CC survivors has become a more significant issue with the increased numbers of survivors^7^.

The Quality of Life (QOL) of patients with CC is an essential assessment for personalizing treatment and providing better care. CC survivors had clinically significant problems with social functioning, constipation, diarrhea, severe lymphedema, menopausal symptoms, reduced body image, sexual or vaginal functioning, as well as difficulties with their finances compared with the general female population. Studies have identified that health-related QOL can also help to predict survival in patients with cancer^8–10^.

There is a dearth of studies focused on the holistic care of the patients with CC primarily on post-treatment long-term QOL in China. It is essential to conduct such studies to identify and address the problem to improve their QOL. The objective of this study was to assess the QOL of the patients with CC after the initial treatment and identify factors that affect the QOL to provide a basis for improved comprehensive clinical care.

## Materials and Methods

### Study design and participants

A descriptive cross-sectional study was conducted after approval by the institutional review board of Zhongnan Hospital of Wuhan University, Department of Gynecological Oncology, Wuhan, Hubei, China. We obtained written informed consent from all the participants. All methods in this study were performed by the relevant guidelines and regulations. A total of 256 patients with CC who visited Zhongnan Hospital from January 2017 to December 2017 and who met the eligibility criteria enrolled in this study. Women with any stage of CC including recurrence (FIGO stage I, II, III and IV), able to understand Chinese and willing to participate in this study were included. Women who declined or who refused to cooperate and patients with psychiatric co-morbidity, communication disorders and or severe other medical condition were excluded from this study.

Treatment guideline for cervical cancer in Zhongnan Hospital is as follows: i) early stage cervical cancer (IA- IIA2) is treated by either surgery and or radiotherapy; ii) advanced stage cervical cancer (IIB – IVA) is treated by primary chemoradiation. However, selected patients with stage IIB are treated with neoadjuvant therapy. Metastatic disease (IVB) primarily treated with chemotherapy. Indications for surgery combined with adjuvant treatment are the presence of one or more pathologic risk factors. Those risk factors are >1/3 stromal invasion, LVSI, and tumor size (i.e., Sedlis criteria) as well as tumor histology of adenocarcinoma and close or positive surgical margins as per National Comprehensive Cancer Network (NCCN) guideline^11^.

### Measurements

The survey instrument consisted of four parts. The first section included demographic information of women, which was collected by interviewing the participant with a structured questionnaire. The second section consisted of clinical characteristics, and it was obtained by reviewing the medical records. The third section was the validated Chinese version of European Organization for Research and Treatment of Cancer Quality of Life Questionnaire Core-30 item (EORTC QLQ-C30) and EORTC QLQ-CX24 (Cervical cancer module)^12–14^. The EORTC questionnaire has been widely employed and tested in different studies among the various cultural group and demonstrated acceptable validity^13,15–21^. The EORTC QLQ-CX24 was selected to assess the QOL of CC patients as it is the most appropriate and valid health-related quality of life cervical cancer specific tool for self-reported evaluation of health status among them^22^. The Chinese version of the EORTC QLQ-CX24 was validated among Chinese cervical cancer patients and reported as a reliable and efficient instrument in clinical research to study QOL^13,21^.

The EORTC QLQ-C30 incorporates five functioning domains (physical, role, cognitive, emotional, and social), three symptom scales (fatigue, pain, and nausea and vomiting), global health and overall QOL scales and six single items that assess additional symptoms commonly reported by cancer patients (dyspnea, appetite loss, sleep disturbance, constipation, and diarrhea) along with perceived financial difficulties^12^. The EORTC QLQ-CX24 includes 24 items consisting of three multi-item scales (symptom experience, body image, and sexual/vaginal functioning scale) and six single-item scales^15^. In the present study, the reliability coefficient for EORTC QLQ-C30 and EORTC QLQ-CX24 was 0.830 and 0.801 respectively.

All scores on the EORTC QLQ-C30 and QLQ-CX24 were transformed into 0 to 100 scale according to the EORTC QLQ scoring manual^23^. Higher scores in GHS and functioning scale represent better levels of functioning and worse levels of symptoms in symptom scales. For EORTC QLQ-CX24, higher scores indicate more symptoms/problems. For the scales sexual activity and sexual enjoyment, a higher score means fewer problems or proper functioning^24^.

### Statistical analysis

For statistical analysis, SPSS (version 16.0) was used. Each scale of EORTC QLQ-C30 and QLQ-CX24 based on QOL scores were dichotomized into problematic and non-problematic. The problematic group was defined as one with a global QOL or a functioning score of 33 or less or with a symptom score of more than 66^23,25^.

Normality tests were carried out for the Global Health Status (GHS), the Functioning Scale, and the Symptom Scale. Data were analyzed with non-parametric tests namely Mann Whitney U test and Kruskal Wallis tests. The patients were divided into groups according to age (<45 years, >46 years), education (illiterate, literate), residence (rural, urban), stage (I, II, III, IV), and treatment modality (surgery only, surgery + radiotherapy + chemotherapy, radiotherapy and or chemotherapy). Multivariable linear regression was performed to explore associations between overall QOL (GHS) and patient and treatment-related variables. A value of P < 0.05 was considered to indicate statistical significance. Clinical relevance was tested to determine statistically significant results regarding (difference of >10 Points)^26^.

## Results

### Sample characteristics

A total of 256 patients with CC were enrolled in the study. The mean age of the patients was 53.4 ± 10.5 years. Almost 44% of the patients reported annual family income less than 1450 (USD). The proportion of patients in FIGO stage I, II, III, and IV were 40.2%, 46.5%, 7.8% and 5.5% respectively. Around 54% of patients had surgery combined with chemo-radiotherapy (Table S1).

### QOL Score

More than half (58.6%) had good global health status, and the majority of the patients had proper functioning in QLQ-C30 functioning scale (range: 71.1% to 89.1%). Financial difficulty was perceived by 53.9% of the patients; insomnia (25.0%) and constipation (21.9%) were the most experienced symptoms. Among the sexually active patients (N = 72, 28.1%), 25% had a problem with sexual enjoyment functioning. Regarding symptoms experienced in QLQ-CX24 scale, 12.5% had menopausal symptoms (Table 1).

**Table 1:** QLQ – C30 & CX24 unadjusted scale scores, the percentage of subjects with problems & in good condition^a^. N = 256.

Variables	Number of items	Mean (SD)	95% C.I.	Scoring <33.3 (%)	Scoring >66.7 (%)
**QLQ -C30 Functional scales** ^**b**^					
Global health status/ QOL	2	65.3 (23.0)	62.2–68.0	4.7	58.6
Physical functioning	5	84.3 (16.8)	82.0–86.4	0	86.7
Role functioning	2	84.4 (22.4)	81.5–87.2	3.1	89.1
Emotional functioning	4	80.3 (18.2)	77.8–82.4	0	84.4
Cognitive functioning	2	80.1 (19.3)	77.7–83.4	0	84.0
Social functioning	2	70.8 (26.9)	67.4–74.0	3.9	71.1
**QLQ-C30 Symptom scales** ^**c**^					
Fatigue	3	24.8 (19.5)	22.4–27.3	58.6	5.5
Nausea & vomiting	2	15.4 (22.5)	12.7–18.4	72.7	4.7
Pain	2	17.7 (20.6)	15.3–20.4	68.8	5.5
Dyspnoea	1	13.3 (19.3)	11.1–15.6	64.8	4.7
Insomnia	1	31.5 (29.3)	28.3–35.0	35.9	25.0
Appetite loss	1	16.9 (24.7)	14.1–19.9	62.5	11.7
Constipation	1	26.3 (30.3)	22.8–30.2	48.4	21.9
Diarrhoea	1	10.2 (18.5)	7.8–12.5	73.4	3.1
Financial difficulties	1	51.6 (37.0)	47.1–56.1	24.2	53.9
**QLQ-CX24 Functional scales** ^**d**^					
Body image	3	25.1 (22.9)	22.3–27.9	86.7	13.3
Sexual activity	1	8.3 (15.6)	6.4–10.3	96.7	3.3
Sexual enjoyment	1	77.8 (25.0)	71.7–82.9	72.7	25.0
Sexual/vaginal functioning	4	75.5 (22.9)	69.6–80.3	73.4	23.4
**QLQ-CX24 Symptom scales** ^**c**^					
Symptom experience	11	14.2 (12.4)	12.8–15.7	92.2	0.8
Lymphoedema	1	8.8 (18.9)	6.8–11.3	78.9	4.7
Peripheral neuropathy	1	22.4 (24.0)	19.7–25.5	46.1	11.7
Menopausal symptoms	1	21.6 (24.5)	18.7–24.7	49.2	12.5
Sexual worry	1	10.4 (19.9)	8.2–13.0	73.4	2.3

^a^For functional scales, subjects scoring <33.3% have problems; those scoring ≥66.7% have good functioning. For symptom scales/symptoms, subjects scoring <33.3% have good functioning; those scoring = 66.7% have problems. ^b^For functional scales, higher scores indicate better functioning. ^c^For symptom scales, higher scores indicate worse functioning. ^d^Higher scores indicate worse functioning, except for Sexual activity and Sexual enjoyment.

### QOL characteristics of patients according to socio-demographic variables

#### Age

Statistically significant and clinically relevant difference was found in two scales of QLQ-CX24; vaginal sexual functioning and peripheral neuropathy were more problematic among the age group of over 46 years. Dyspnea (p = 0.000), and sexual worry (p = 0.002) were significant among the patients under 45 years (Table 2).

**Table 2:** Quality of life score by age, education, and residence of the patients.

QLQ Items	Age		P	Clrel*	Education		P	Clrel*	Residence		P	Clrel*
	≤45	≥46			Illiterate	Literate			Rural	Urban		
	N = 66	N = 190			N = 56	N = 200			N = 126	N = 130		
**C30 Functional scales**												
Global health status/ QOL	68.7 ± 25.0	64.1 ± 22.2	0.073	No	53.5 ± 23.4	68.5 ± 21.8	0.000	Yes	55.1 ± 24.9	73.3 ± 18.6	0.000	Yes
Physical functioning	86.9 ± 22.2	83.4 ± 17.6	0.291	No	79.5 ± 19.0	85.6 ± 15.8	0.042	No	80.6 ± 21.3	86.5 ± 14.8	0.178	No
Role functioning	86.4 ± 16.8	83.7 ± 24.0	0.942	No	82.1 ± 24.2	85.0 ± 21.8	0.367	No	81.1 ± 23.6	84.4 ± 23.0	0.231	No
Emotional functioning	81.3 ± 15.3	79.9 ± 19.1	0.984	No	79.4 ± 20.7	80.5 ± 17.4	0.907	No	78.2 ± 20.8	81.4 ± 15.9	0.595	No
Cognitive functioning	83.8 ± 15.7	78.8 ± 20.3	0.139	No	78.6 ± 20.0	70.5 ± 18.8	0.651	No	79.2 ± 19.2	80.4 ± 20.4	0.553	No
Social functioning	75.2 ± 24.5	69.3 ± 27.7	0.158	No	58.3 ± 33.6	74.3 ± 24.2	0.001	Yes	59.1 ± 0.8	74.8 ± 22.5	0.001	Yes
**Symptom scales**												
Fatigue	22.6 ± 16.2	25.6 ± 20.6	0.470	No	25.8 ± 18.9	24.5 ± 19.7	0.352	No	25.5 ± 22.0	25.4 ± 18.0	0.696	No
Nausea & vomiting	11.1 ± 14.7	16.8 ± 24.5	0.365	No	20.8 ± 25.7	13.8 ± 21.4	0.040	No	20.8 ± 26.5	13.3 ± 22.5	0.023	No
Pain	17.2 ± 15.2	17.9 ± 22.2	0.411	No	22.0 ± 24.2	16.5 ± 19.4	0.233	No	21.6 ± 25.9	15.2 ± 19.3	0.179	No
Dyspnoea	20.2 ± 20.1	10.9 ± 18.4	0.000	No	9.5 ± 15.1	14.3 ± 20.1	0.165	No	10.6 ± 17.1	15.5 ± 21.9	0.164	No
Insomnia	26.2 ± 28.5	33.3 ± 29.5	0.069	No	29.8 ± 28.9	32.0 ± 29.5	0.643	No	26.5 ± 27.3	31.8 ± 27.3	0.174	No
Appetite loss	11.1 ± 19.7	18.9 ± 25.9	0.033	No	28.5 ± 28.0	13.7 ± 22.7	0.000	Yes	20.4 ± 26.9	14.8 ± 26.0	0.116	No
Constipation	20.2 ± 24.7	28.4 ± 31.7	0.750	No	32.1 ± 34.2	24.7 ± 28.9	0.174	No	28.8 ± 32.4	23.7 ± 30.5	0.291	No
Diarrhoea	12.1 ± 23.1	9.5 ± 16.5	0.748	No	8.3 ± 14.5	10.7 ± 19.4	0.655	No	8.3 ± 14.5	14.8 ± 22.9	0.079	No
Financial difficulties	46.5 ± 35.0	53.3 ± 37.7	0.179	No	67.8 ± 38.6	47.0 ± 35.4	0.000	Yes	72.7 ± 32.2	42.2 ± 36.3	0.000	Yes
**CX24 Functional scales**												
Body image	21.5 ± 21.7	26.3 ± 23.1	0.118	No	29.4 ± 25.5	23.9 ± 21.9	0.192	No	24.7 ± 23.3	24.4 ± 21.6	0.888	No
Sexual activity	8.5 ± 15.8	8.2 ± 15.6	0.863	No	4.2 ± 11.1	9.5 ± 16.4	0.027	No	7.6 ± 14.9	8.5 ± 16.2	0.756	No
Sexual enjoyment (n = 80)	70.4 ± 19.4	80.2 ± 26.3	0.199	No	55.6 ± 40.8	68.1 ± 41.2	0.191	No	55.5 ± 36.1	70.2 ± 45.0	0.018	Yes
Sexual/vaginal functioning (n = 72)	86.1 ± 13.4	71.9 ± 24.3	0.025	Yes	87.5 ± 14.8	73.9 ± 23.4	0.099	No	76.0 ± 14.5	71.1 ± 28.6	0.865	No
**Symptom scales**												
Symptom experience	10.5 ± 7.5	15.9 ± 13.6	0.013	No	15.9 ± 12.5	13.7 ± 12.4	0.209	No	17.1 ± 12.0	15.4 ± 15.2	0.083	No
Lymphoedema	10.1 ± 19.4	8.4 ± 18.7	0.457	No	11.9 ± 22.4	8.0 ± 17.7	0.147	No	15.9 ± 24.2	3.7 ± 12.7	0.000	Yes
Peripheral neuropathy	14.1 ± 18.5	25.3 ± 25.0	0.002	Yes	26.2 ± 27.5	21.3 ± 22.9	0.310	No	26.5 ± 27.3	19.2 ± 22.9	0.081	No
Menopausal symptoms	24.2 ± 27.7	20.7 ± 23.3	0.510	No	20.2 ± 24.4	22.0 ± 24.6	0.614	No	25.0 ± 25.9	20.0 ± 24.9	0.158	No
Sexual worry	14.1 ± 16.6	9.1 ± 20.8	0.002	No	3.6 ± 10.4	12.3 ± 21.5	0.002	No	8.3 ± 19.0	13.3 ± 23.8	0.101	No

*Clinical relevance ≥10 points differences.

#### Education

Literate patients had good global/QOL (p = 0.000, clinically relevant) and social functioning (p = 0.001, clinically relevant), physical functioning (p = 0.042). Illiterate patient’s data showed more appetite loss, more financial difficulties (p = 0.000, clinically relevant) and more nausea/vomiting (p = 0.040) (Table 2).

#### Residence

Patients living in an urban area showed better global/QOL (p = 0.000, clinically relevant), good social functioning (p = 0.001, clinically relevant). Patients living in a rural area reported financial difficulties (p = 0.000, clinically relevant), problems in sexual enjoyment (p = 0.018, clinically relevant), and lymphoedema (p = 0.000, clinically relevant) (Table 2).

#### Occupation

Service holder showed good global QOL. However, retired/unemployed/housewife group of patients had good social functioning (p = 0.000, clinically relevant) (Table 3).

**Table 3:** Quality of life score according to occupation and family annual income.

QLQ Items	Occupation			P	Clrel*	Family income		P	Clrel*
	Agriculture	Employed	Others^a^			≤1450 USD	>1451 USD		
	N = 88	N = 90	N = 78			N = 112	N = 144		
**C30 Functional scales**									
Global health status/ QOL	55.1 ± 24.9	73.3 ± 18.7	67.5 ± 21.1	0.000	Yes	57.7 ± 25.0	71.2 ± 19.4	0.000	Yes
Physical functioning	80.6 ± 21.3	86.5 ± 14.8	85.8 ± 11.9	0.398	No	82.4 ± 19.2	85.7 ± 14.5	0.496	No
Role functioning	81.9 ± 22.0	84.4 ± 23.0	88.0 ± 19.7	0.107	No	80.6 ± 24.2	87.3 ± 20.4	0.017	No
Emotional functioning	78.2 ± 20.8	81.4 ± 15.9	81.2 ± 19.7	0.775	No	78.1 ± 9.2	81.9 ± 17.3	0.136	No
Cognitive functioning	79.2 ± 19.3	80.4 ± 20.4	80.8 ± 18.2	0.817	No	78.6 ± 19.6	81.2 ± 18.9	0.266	No
Social functioning	59.1 ± 30.8	74.8 ± 22.5	79.5 ± 22.3	0.000	Yes	60.4 ± 28.9	78.9 ± 22.3	0.000	Yes
**C30 Symptom scales**									
Fatigue	25.5 ± 22.0	25.4 ± 18.0	23.4 ± 18.4	0.683	No	27.6 ± 21.4	22.7 ± 17.7	0.094	No
Nausea & vomiting	20.8 ± 26.5	13.3 ± 22.5	11.5 ± 15.7	0.037	No	16.7 ± 24.5	14.3 ± 20.9	0.591	No
Pain	21.6 ± 25.9	15.2 ± 19.3	16.2 ± 13.9	0.322	No	19.3 ± 24.0	16.4 ± 17.5	0.917	No
Dyspnoea	10.6 ± 17.2	15.5 ± 21.9	13.7 ± 16.2	0.325	No	13.1 ± 19.7	13.4 ± 19.0	0.785	No
Insomnia	26.5 ± 27.3	31.8 ± 27.3	36.7 ± 32.9	0.126	No	29.7 ± 28.8	32.9 ± 29.7	0.394	No
Appetite loss	20.4 ± 26.9	14.8 ± 26.0	15.4 ± 19.9	0.255	No	17.3 ± 25.3	16.7 ± 24.3	0.956	No
Constipation	28.8 ± 32.4	23.7 ± 30.5	26.5 ± 27.6	0.484	No	29.2 ± 31.0	24.1 ± 29.6	0.170	No
Diarrhoea	8.3 ± 14.5	14.8 ± 22.9	6.8 ± 15.5	0.025	No	12.5 ± 20.6	8.3 ± 16.5	0.078	No
Financial difficulties	72.7 ± 32.2	42.2 ± 36.3	38.5 ± 32.7	0.000	Yes	68.5 ± 32.5	38.4 ± 35.1	0.000	Yes
**CX24 Functional scales**									
Body image	24.7 ± 23.3	24.4 ± 21.6	26.2 ± 24.1	0.912	No	24.9 ± 24.4	25.2 ± 21.7	0.640	No
Sexual activity	7.5 ± 14.9	8.5 ± 16.2	8.9 ± 15.8	0.831	No	6.5 ± 14.7	9.7 ± 16.2	0.071	No
Sexual enjoyment (n = 80)	55.5 ± 36.1	70.2 ± 45.0	69.4 ± 37.9	0.052	No	55.6 ± 41.3	72.3 ± 40.1	0.021	Yes
Sexual/vaginal functioning (n = 72)	76.0 ± 14.5	71.1 ± 28.6	81.8 ± 16.2	0.465	No	75.7 ± 27.9	75.3 ± 20.3	0.493	No
**CX24 Symptom scales**									
Symptom experience	17.1 ± 12.1	15.4 ± 15.3	9.5 ± 6.6	0.000	No	15.6 ± 11.1	13.1 ± 13.2	0.010	No
Lymphoedema	15.9 ± 24.2	3.7 ± 12.7	6.8 ± 15.5	0.000	Yes	10.7 ± 19.0	7.4 ± 18.7	0.058	No
Peripheral neuropathy	26.5 ± 27.3	19.2 ± 22.9	21.4 ± 20.8	0.196	No	23.8 ± 25.8	21.3 ± 22.5	0.590	No
Menopausal symptoms	25 ± 25.9	20.0 ± 24.9	19.6 ± 22.4	0.296	No	23.2 ± 24.4	20.4 ± 24.6	0.278	No
Sexual worry	8.3 ± 19.0	13.3 ± 23.8	9.4 ± 15.1	0.250	No	8.9 ± 18.4	11.6 ± 20.9	0.278	No

*Clinical relevance ≥10 points differences. ^a^Unemployed/retired/housewife.

#### Family income

Patients with an annual family income more than US $1451 showed better global/QOL (p = 0.000, clinically relevant), better social functioning (p = 0.034, clinically relevant) and role functioning (p = 0.003) than with the lower income group (Table 3).

#### Stage of cancer

Table 4 showed that patients diagnosed in stage I had better global/QOL, better physical (p = 0.003), role (p = 0.003), social functioning (p = 0.034); more problems like fatigue (p = 0.036), nausea and vomiting (p = 0.009), appetite loss (p = 0.000) and financial difficulties (p = 0.000) were experienced by patients diagnosed in stage IV. These all findings were clinically relevant (Table 4).

**Table 4:** Quality of life score according to the stage of cervical cancer and treatment modalities.

**QLQ Items**	**Stage**				P	Clrel*	**Treatment**			P	Clrel*
	**I**	**II**	**III**	**IV**			**Surgery**	**S + C + R**	**C/R**		
	**N = 103**	**N = 119**	**N = 20**	**N = 14**			**N = 60**	**N = 137**	**N = 59**		
**C30 Functional scales**											
Global health status/ QOL	70.5 ± 21.8	66.1 ± 21.0	55.8 ± 24.2	33.3 ± 17.9	0.000	Yes	75.8 ± 18.6	66.8 ± 22.9	51.1 ± 20.6	0.000	Yes
Physical functioning	87.7 ± 21.8	83.5 ± 22.1	85.0 ± 20.9	66.7 ± 24.4	0.003	Yes	89.8 ± 16.9	83.0 ± 16.0	81.6 ± 17.4	0.000	No
Role functioning	87.7 ± 21.8	83.5 ± 22.1	85.0 ± 20.9	66.7 ± 24.4	0.003	Yes	91.7 ± 16.1	82.7 ± 24.0	80.8 ± 22.5	0.003	Yes
Emotional functioning	79.5 ± 18.9	81.4 ± 16.9	80.8 ± 20.2	75.0 ± 21.2	0.714	No	79.4 ± 19.9	80.6 ± 17.3	80.2 ± 18.6	0.991	No
Cognitive functioning	80.7 ± 17.2	79.4 ± 20.5	85.0 ± 19.4	73.8 ± 22.4	0.413	No	81.1 ± 19.9	78.1 ± 19.2	83.6 ± 18.4	0.124	No
Social functioning	76.4 ± 25.3	68.9 ± 25.8	61.7 ± 31.6	59.5 ± 34.4	0.034	Yes	81.7 ± 21.8	70.9 ± 25.6	59.6 ± 30.5	0.000	Yes
**C30 Symptom scales**											
Fatigue	22.6 ± 21.7	24.6 ± 16.4	28.9 ± 21.1	36.5 ± 22.8	0.036	Yes	20.4 ± 22.2	25.7 ± 19.0	27.3 ± 17.3	0.014	No
Nausea & vomiting	9.2 ± 15.2	18.3 ± 24.3	20.0 ± 25.1	28.6 ± 35.5	0.009	Yes	6.7 ± 16.0	14.5 ± 17.8	26.3 ± 32.0	0.000	Yes
Pain	15.8 ± 22.4	17.9 ± 18.3	23.3 ± 20.5	21.4 ± 25.7	0.166	No	11.1 ± 20.9	18.4 ± 19.4	22.9 ± 21.6	0.000	Yes
Dyspnoea	11.6 ± 18.5	15.1 ± 19.8	6.7 ± 13.7	19.0 ± 25.1	0.174	No	6.7 ± 16.0	16.0 ± 20.2	13.6 ± 18.7	0.003	No
Insomnia	31.7 ± 31.1	30.2 ± 28.4	30.0 ± 23.9	42.8 ± 30.4	0.446	No	25.6 ± 22.4	31.6 ± 32.7	37.3 ± 26.3	0.077	No
Appetite loss	8.7 ± 18.6	19.3 ± 23.1	26.7 ± 29.8	42.8 ± 40.1	0.000	Yes	4.4 ± 11.4	18.5 ± 25.2	25.9 ± 28.4	0.000	Yes
Constipation	19.7 ± 28.2	29.4 ± 30.1	36.7 ± 32.3	33.3 ± 36.9	0.016	Yes	15.6 ± 28.4	27.7 ± 29.0	33.9 ± 32.4	0.001	Yes
Diarrhoea	7.8 ± 15.6	10.0 ± 16.5	16.7 ± 31.5	19.0 ± 25.2	0.221	No	4.4 ± 11.4	10.4 ± 18.4	15.2 ± 22.6	0.006	Yes
Financial difficulties	41.1 ± 35.6	55.2 ± 35.9	63.3 ± 35.7	80.9 ± 36.3	0.000	Yes	33.3 ± 36.8	54.2 ± 33.6	63.8 ± 38.8	0.000	Yes
**CX24 Functional scales**											
Body image	22.9 ± 23.9	26.0 ± 22.3	31.1 ± 25.4	24.6 ± 14.6	0.286	No	22.4 ± 23.5	25.1 ± 21.2	27.9 ± 25.8	0.391	No
Sexual activity	7.1 ± 15.9	8.9 ± 15.5	6.7 ± 13.7	14.3 ± 17.1	0.216	No	7.2 ± 16.3	8.2 ± 15.0	9.6 ± 16.4	0.561	No
Sexual enjoyment (n = 80)	73.0 ± 35.5	52.4 ± 50.8	83.3 ± 19.2	77.8 ± 17.2	0.334	No	74.5 ± 33.9	58.1 ± 47.3	69.7 ± 37.9	0.429	No
Sexual/vaginal functioning (n = 72)	75.4 ± 26.2	69.7 ± 17.5	95.8 ± 4.8	83.3 ± 14.9	0.054	No	85.9 ± 13.9	60.0 ± 24.4	88.3 ± 13.1	0.000	Yes
**CX24 Symptom scales**											
Symptom experience	12.7 ± 9.3	15.6 ± 15.1	14.2 ± 10.5	13.2 ± 9.0	0.901	No	10.9 ± 7.1	14.9 ± 14.0	15.7 ± 12.4	0.161	No
Lymphoedema	8.4 ± 20.7	8.9 ± 17.2	6.7 ± 13.7	14.3 ± 25.2	0.654	No	10.0 ± 21.5	8.7 ± 18.6	7.9 ± 16.8	0.974	No
Peripheral neuropathy	21.4 ± 21.3	24.1 ± 24.9	16.7 ± 17.0	23.8 ± 40.1	0.582	No	20.0 ± 23.9	23.8 ± 22.8	21.5 ± 26.8	0.345	No
Menopausal symptoms	24.6 ± 27.6	21.3 ± 22.4	13.3 ± 16.7	14.3 ± 25.2	0.221	No	22.2 ± 27.9	22.9 ± 23.8	18.1 ± 22.6	0.400	No
Sexual worry	16.2 ± 20.3	5.0 ± 16.0	16.7 ± 31.5	4.7 ± 12.1	0.000	Yes	14.4 ± 16.6	8.2 ± 17.5	11.3 ± 26.7	0.007	No

*Clinical relevance ≥10 points differences; S + C + R: surgery + chemotherapy + radiotherapy; C/R: chemotherapy and or radiotherapy.

#### Treatment modalities

Patients who underwent surgery showed better global/QOL (p = 0.000, clinically relevant), better role (p = 0.003, clinically relevant), and social functioning (p = 0.000). Regarding symptom scales patients with chemotherapy and/or radiotherapy tend to have more problem with nausea and vomiting (p = 0.000), pain (p = 0.000), appetite loss (p = 0.000), constipation (p = 0.001), diarrhea (p = 0.006), and financial difficulties (p = 0.000), which were clinically relevant too (Table 4).

#### Time since diagnosis

**Figure 1:**
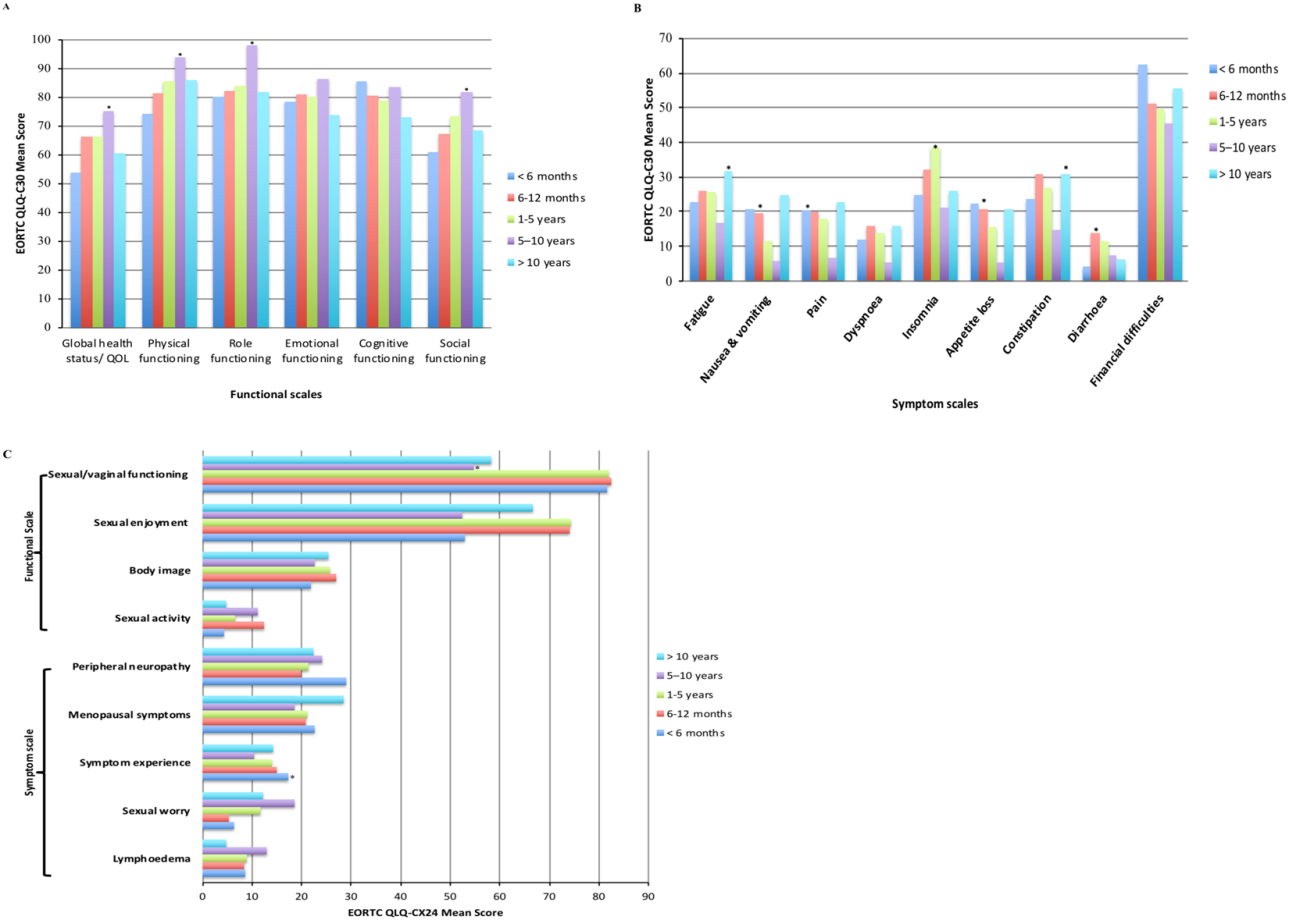
Quality of life scores of cervical cancer patients according to time since diagnosis. (**A**) EORTC QLQ-C30- functional scores. (**B**) EORTC QLQ-C30- symptoms scores (**C**). EORTC QLQ-CX24 scores.

Patients with 5 to 10 years of survival reported good global QOL, physical, role, social and sexual and vagina functioning (p < 0.05, clinically relevant). However, most symptoms, pain (p = 0.006, clinically relevant), nausea/vomiting (p = 0.003, clinically relevant) and appetite loss (p = 0.015, clinically relevant), were experienced within 12 months. Patients who were diagnosed more than ten years reported more fatigue (p = 0.045, clinically relevant) and constipation (p = 0.041, clinically relevant) symptoms (Fig. 1).

#### Multiple linear regressions

Table 5 presents the association between overall QOL and different variables related to patient and treatment. It shows that lower family income, the rural area of living had a negative impact on the overall QOL, and the advanced stage of cancer had a statistically significant effect on overall QOL of patients (Table 5).

**Table 5:** Multiple linear regression of overall QOL.

Variables	Unstandardized coefficients (B)	Coefficients (Beta)	t-statistics	p
Constant	52.435		4.043	0.000
Age	1.578	0.027	0.397	0.692
Family annual income	10.741	0.225	2.862	0.005
Stage of cancer	−7.666	−0.273	−3.326	0.001
Treatment modality	−2.846	−0.088	−1.072	0.285
Time since diagnosis	−0.346	−0.017	−0.253	0.800
Residence	8.120	0.171	1.999	0.047
Education status	0.647	0.012	0.151	0.880

Note: Adjusted R^2^ = 0.29.

## Discussion

The characteristics of patients with CC and QOL after treatment was the focus of this study. More than half of the patients with CC reported an excellent QOL, similar to the most published data^21,27^. Consistent with our finding, the study reported that global health status of CC patients was 59.5 ± 10.9 in India^28^. Regarding functional dimension, the patients reported proper functioning in overall scales (score range: 70.8 ± 26.9 to 91.7 ± 15.6) except for sexual enjoyment and sexual or vaginal functioning. The higher scores might be because nearly 60% of the patients enrolled in our study had early-stage cancer (stage IA-IIA). The adverse symptoms experienced mostly were insomnia, constipation, appetite loss, financial difficulty, menopausal symptoms and peripheral neuropathy. These findings are resembling other similar studies^10,21^.

Patient’s lower annual family income and rural area of living showed a negative impact on global QOL. Reports indicate that less education had been associated with limited knowledge about health issues and poor health^10,21^. Cancer survivors living in the rural area are at higher risk for a variety of poor health outcomes^29^ and poor socioeconomic status (e.g., lower education level and income) are less likely to have follow-up care with providers leading to poor health outcome^27,30^. Patients from a rural area or with lower economic status or illiterate people might be unaware of cervical cancer. So, these individuals may usually reach the hospital with the late stage of cancer, which leads to poor treatment outcome and consequently a reduced quality of life. Therefore, these problems should be given due attention by the concerned authority to improve the QOL.

Younger patients reported better functional scales than older age groups which are similar to the study result reported by the Action Study Group^27^. Wenzel *et al*. reported that younger CC patients experience a persistent detrimental effect on their QOL^31^. Several studies reported a negative impact of sexuality across all CC patients^24,32–34^. Young patients in our study had reported more sexual worry compared to an older group of patients. In line with our study, the previous research said younger patients were concerned with fertility, femininity, treatment-related menopause, and relationship issues^35^. Cervical cancer is known as the human papillomavirus (HPV) related cancer, and a positive high-risk HPV regarded as a sexually transmitted infection (STI). Young people are sexually active, so the chances of having STI is also higher among those who have risky sexual behavior; on the other hand, they have access to the information about STI or HPV which might be partially correct. Therefore, many young women especially the educated patients might blame their husband or partner for the disease, which leads to relationship problems and causes more sexual worry. Many studies reported a negative impact on sexuality among patients with CC and its treatment^21,34,36^. These findings underline the importance of counseling regarding these problems, especially with younger and educated patients about the right information of high-risk HPV infection and the existence of all other co-factors as well.

Sexuality is an essential aspect of gynecological cancer, thus being a crucial determinant of QOL. In the present study, there was a significant decrease in sexual enjoyment and the sexual and vaginal functioning score. Previous reports also stated that 40% to 100% of individuals face sexual dysfunction after treatments because CC and its treatment affect the same areas of the body that are involved in sexual response^10,37^. Patients with surgery along with chemotherapy and radiotherapy reported worse in sexual and vaginal functioning than those with surgery. Sexual dysfunction from surgery is mainly due to the shortened vagina, vaginal dryness, decreased libido^38,39^. However, after radiotherapy, sexual dysfunction is caused by vaginal stenosis which leads to dyspareunia, difficulty in orgasm, a decrease in sexual satisfaction, and changes in body image^40^.

Patients who had undergone radiotherapy and chemotherapy had experienced more symptoms like fatigue, nausea and vomiting, pain, appetite loss, constipation, diarrhea and financial difficulty than those who underwent surgery only. Many studies have mentioned that radiotherapy to the pelvic cavity has easily caused intestinal dysfunctions^41^. Radiation has also created lactose and bile salt malabsorption, intestinal bacteria imbalance and altered intestinal peristalsis. Therefore, radiotherapy for CC often causes intestinal dysfunctions^42,43^.

The advanced stage of cancer showed a negative impact on global QOL and patients with early-stage cancer reported better QOL. Several studies reported that, for global health status or overall QOL, patients with stage I, II, and III of cancer have higher QOL compared to stage IV^10,44^. Regarding role functioning, patients in stage I had the better QOL followed by stage II; stage IV had the worse role functioning. Patients at the late stage of cancer would have poor role functioning as these patients usually planned for palliative management and therefore unable to perform much work.

Also, time since diagnosis affects the self-reported health status and QOL among cancer survivors^9^. The present study findings are consistent that time is a significant factor in QOL of survivors. Patients diagnosed for 5 to 10 years reported higher scores on global QOL, physical functioning, role functioning and sexual and vaginal functioning. However, after ten years since diagnosis, the functional scale (global QoL, physical, role, social and sexual and vaginal functioning) scores were decreasing than in the 5–10 years’ period and also nearly similar to the time of 6–12 months after diagnosis. Similarly, fatigue, nausea and vomiting, pain and constipation symptoms were increased with more than ten years of survivorship. These findings could be the result of the long-term effect of chemotherapy and radiotherapy that patients experience bowel, bladder, and sexual dysfunction even after many years of treatment is in line with another study^45^. To improve the health outcome of CC patients the treatment and management should focus on time since diagnosis as well. Patients need long-term care for better health outcome. This finding suggests that the QOL of the patient is changing over the long term. Further study is recommended to evaluate the QOL of long-term survivors.

Nevertheless, this study has some limitations. The QOL of cancer survivors changes over time. As this is a cross-sectional design, the assessment of QOL was not done over time, and the lack of the comparison of QOL score before and after treatment contribute to the limitations. The data collection was limited to a single institution based so the study results could not be generalized to the whole population of cancer survivors in China. However, the study contributes to how to improve patient care and further research for women with CC in China. Longitudinal and intervention studies with control group may better evaluate the QOL of CC survivors.

## Conclusion

More than half of the patients with cervical cancer reported an excellent QOL. The rural area of living and poor economic status of the patients with cervical cancer has a negative impact on overall quality of life. Younger and educated patients are more worried about sexuality and patients treated with multiple therapies had more problems with their QOL scales than patients treated with surgery only.

## Electronic supplementary material

Supplementary Table 1

## References

[1] Ferlay, J. *et al*. GLOBOCAN 2012: Estimated Cancer Incidence, Mortality and Prevalence Worldwide in 2012. *GLOBOCAN 2012v1.0* at http://globocan.iarc.fr/Pages/fact_sheets_cancer.aspx (2013).

[2] Chen W (2016). Cancer Statistics in China. CA Cancer J. Clin..

[3] Cai HB (2010). Trends in cervical cancer in young women in Hubei, China. Int. J. Gynecol. Cancer.

[4] Zhang L (2012). Prevalence and type distribution of high-risk human papillomavirus infections among women in Wufeng County, China. Arch. Gynecol. Obstet..

[5] National Comprehensive Cancer Network. *NCCN Clinical Practice Guideline in Oncology: Cervical cancer*, 10.1017/CBO9781139046947.057 (2016).

[6] Zhou W (2016). Survey of cervical cancer survivors regarding quality of life and sexual function. J. Cancer Res. Ther..

[7] Ye S, Yang J, Cao D, Lang J, Shen K (2014). A systematic review of quality of life and sexual function of patients with cervical cancer after treatment. Int. J. Gynecol. Cancer.

[8] Quinten C (2009). Baseline quality of life as a prognostic indicator of survival: a meta-analysis of individual patient data from EORTC clinical trials. Lancet Oncol..

[9] Kim M-K (2016). Health-Related Quality of Life and Sociodemographic Characteristics as Prognostic Indicators of Long-term Survival in Disease-Free Cervical Cancer Survivors. Int. J. Gynecol. Cancer.

[10] Park SY (2007). Quality of life and sexual problems in disease-free survivors of cervical cancer compared with the general population. Cancer.

[11] National Comprehensive Cancer Network. *NCCN Clinical Practice Guideline in Oncology (NCCN Guidelines) Cervical cancer. NCCN*, 10.1017/CBO9781139046947.057 (2018).

[12] Fayers, P. *et al*. *EORTC QLQ-C30 Scoring Manual*. (EORTC, 2001).

[13] Hua CH (2013). Validation of the European Organization for Research and Treatment of Cancer cervical cancer module for Chinese patients with cervical cancer. Patient Prefer. Adherence.

[14] Wan C (2008). Validation of the simplified Chinese version of EORTC QLQ-C30 from the measurements of five types of inpatients with cancer. Ann. Oncol..

[15] Greimel ER (2006). The European Organization for Research and Treatment of Cancer (EORTC) Quality-of-Life questionnaire cervical cancer module: EORTC QLQ-CX24. Cancer.

[16] Jayasekara H, Rajapaksa LC, Greimel ER (2008). The EORTC QLQ-CX24 cervical cancer-specific quality of life questionnaire: psychometric properties in a South Asian sample of cervical cancer patients. Psychooncology..

[17] Paradowska D (2014). Validation of the Polish version of the EORTC QLQ-CX24 module for the assessment of health-related quality of life in women with cervical cancer. Eur. J. Cancer Care (Engl)..

[18] Shin DW (2009). Cross-Cultural Application of the Korean Version of the European Organization for Research and Treatment of Cancer Quality of Life Questionnaire Cervical Cancer Module. Oncology.

[19] Singer S (2010). Patients’ acceptance and psychometric properties of the EORTC QLQ-CX24 after surgery. Gynecol. Oncol..

[20] du Toit GC, Kidd M (2016). An analysis of the psychometric properties of the translated versions of the European Organisation for the Research and Treatment of Cancer QLQ CX24 questionnaire in the two South African indigenous languages of Xhosa and Afrikaans. Eur. J. Cancer Care (Engl)..

[21] Huang H-Y (2017). Quality of life of breast and cervical cancer survivors. BMC Womens. Health.

[22] Tax C, Steenbergen ME, Zusterzeel PLM, Bekkers RLM, Rovers MM (2017). Measuring health-related quality of life in cervical cancer patients: a systematic review of the most used questionnaires and their validity. BMC Med. Res. Methodol..

[23] Fayers, P. M. *et al*. EORTC QLQ-C30 Scoring Manual. at http://www.forskningsdatabasen.dk/en/catalog/2192871649 (2001).

[24] Bjelic-Radisic V (2012). Quality of life characteristics inpatients with cervical cancer. Eur. J. Cancer.

[25] Ahn SH (2007). Health-related quality of life in disease-free survivors of breast cancer with the general population. Ann. Oncol..

[26] Osoba D (2002). A taxonomy of the uses of health related-quality of life instruments in cancer care and the clinical meaningfulness of the results. Med. Care.

[27] The Action Study Group. Health-related quality of life and psychological distress among cancer survivors in Southeast Asia: results from a longitudinal study in eight low- and middle-income countries. *BMC Med*. **15**, 10 (2017). 10.1186/s12916-016-0768-2 PMC5234136 28081724

[28] Dahiya N (2016). Quality of Life of Patients with Advanced Cervical Cancer before and after Chemoradiotherapy. Asian Pac. J. Cancer Prev..

[29] Weaver KE, Geiger AM, Lu L, Case LD (2013). Rural-urban disparities in health status among US cancer survivors. Cancer.

[30] DiMartino LD, Birken SA, Mayer DK (2017). The Relationship Between Cancer Survivors’ Socioeconomic Status and Reports of Follow-up Care Discussions with Providers. J. Cancer Educ..

[31] Wenzel L (2005). Quality of life in long-term cervical cancer survivors. Gynecol. Oncol..

[32] Chia-Chun L, Ting-Chang C, Yun-Fang T, Lynn C (2017). Quality of life among survivors of early-stage cervical cancer in Taiwan: an exploration of treatment modality differences. Qual. Life Res..

[33] Muliira R, Salas A, O’Brien B (2017). Quality of life among female cancer survivors inAfrica: An integrative literature review. Asia-Pacific J. Oncol. Nurs..

[34] Derks M (2017). Long-Term Morbidity and Quality of Life in Cervical Cancer Survivors. Int. J. Gynecol. Cancer.

[35] Chan YM (2001). A longitudinal study on quality of life after gynecologic cancer treatment. Gynecol. Oncol..

[36] Sung UL (2017). General health status of long-term cervical cancer survivors after radiotherapy. Strahlentherapie und Onkol..

[37] Kumar, S. *et al*. PrediQt-Cx: Post treatment health related quality of life prediction model for cervical cancer patients. *PLoS One* **9** (2014). 10.1371/journal.pone.0089851 PMC3935936 24587074

[38] Burns M, Costello J, Ryan-Woolley B, Davidson S (2007). Assessing the impact of late treatment effects in cervical cancer: An exploratory study of women’s sexuality: Original article. Eur. J. Cancer Care (Engl)..

[39] Jensen PT (2004). Early-Stage Cervical Carcinoma, Radical Hysterectomy, and Sexual Function: A Longitudinal Study. Cancer.

[40] Buković D (2003). Sexual life after cervical carcinoma. Coll. Antropol..

[41] Pieterse QD (2013). Self-reported sexual, bowel and bladder function in cervical cancer patients following different treatment modalities: Longitudinal prospective cohort study. Int. J. Gynecol. Cancer.

[42] Stacey R, Green JT (2013). Radiation-induced small bowel disease: Latest developments and clinical guidance. Ther. Adv. Chronic Dis..

[43] Steen R, Dahl AA, Hess SL, Kiserud CE (2017). A study of chronic fatigue in Norwegian cervical cancer survivors. Gynecol. Oncol..

[44] Xie Y (2013). Assessment of quality of life for the patients with cervical cancer at different clinical stages. Chin. J. Cancer.

[45] Pfaendler KS, Wenzel L, Mechanic MB, Penner KR (2015). Cervical cancer survivorship: Long-term quality of life and social support HHS Public Access. Clin Ther. January.

