# Supplementary material for: Impact of cervical cancer on quality of life of women in Hubei, China
Source: Sci Rep. 2018 Aug 10;8:11993. doi: 10.1038/s41598-018-30506-6 (PMC6086893; doi:10.1038/s41598-018-30506-6)
Supplement: Supplementary file 1 — Supplementary Table 1 [file 41598_2018_30506_MOESM1_ESM.docx]

**Impact of cervical cancer on quality of life of women in Hubei, China**

Niresh Thapa ^1,2, +^, Muna Maharjan ^3,+^, Yan Xiong ^1^, Daqiong Jiang ^1^, Thi-Phuong Nguyen ^1^, Marcia A. Petrini ^4^, and Hongbing Cai ^1, *^

^1^ Department of Gynecological Oncology, Zhongnan Hospital of Wuhan University, Hubei Cancer Clinical Study Center, Hubei Key Laboratory of Tumor Biological Behaviors, Wuhan, Hubei, China

^2^ Karnali Academy of Health Sciences, Jumla, Nepal

^3^ Zhongnan Hospital of Wuhan University, Hope School of Nursing, Wuhan, Hubei, China

^4^ Faculty of Nursing, Chiang Mai University, Chiang Mai, Thailand

^+^ These authors contributed equally to this study.

* Corresponding author at Department of Gynecological Oncology, Zhongnan Hospital of Wuhan University, Hubei Cancer Clinical Study Center, Hubei Key Laboratory of Tumor Biological Behaviors, Wuhan, Hubei 430071, China.

Email address: [caihongbing2105@outlook.com](mailto:caihongbing2105@outlook.com) (Hongbing Cai)

**Table S1 Socio-demographic and clinical characteristics of the participants N= 256**

| Variables | Frequency (%) |
| --- | --- |
| **Age** |  |
| 30-45 | 66 (25.8) |
| 46-60 | 126 (49.2) |
| > 61 | 64 (25.0) |
| Mean age | 53.4 + 10.5 |
| **Number of children** |  |
| 0 | 18 (7.0) |
| 1 | 96 (37.5) |
| 2 | 96 (37.5) |
| > 3 | 46 (18.0) |
| **Education status** |  |
| Illiterate | 56 (21.9) |
| Primary and secondary | 120 (46.9) |
| Higher secondary | 52 (20.3) |
| University level | 28 (10.9) |
| **Occupation** |  |
| Service | 90 (35.2) |
| Agriculture | 88 (34.4) |
| Unemployed/ retired/ homemaker | 78 (30.5) |
| **Family annual income (USD)** |  |
| < 1450 | 112 (43.8) |
| >1450 | 144 (56.3) |
| **Smoking history** |  |
| Smokers | 22 (8.6) |
| Non-smokers | 234 (91.4) |
| **Family history of cancer** |  |
| Positive | 38 (14.8) |
| Negative | 218 (85.2) |
| **Stage of Cancer** |  |
| Stage I | 103 (40.2) |
| Stage II | 119 (46.5) |
| Stage III | 20 (7.8) |
| Stage IV | 14 (5.5) |
| **Treatment** |  |
| Surgery only | 60 (23.5) |
| Surgery + Radiotherapy + Chemotherapy | 137 (53.5) |
| Radiotherapy + Chemotherapy | 59 (23.0) |
| **Co-morbidity (multiple response)** |  |
| Diabetes | 24 (9.4) |
| Hypertension | 38 (14.8) |
| None | 186 (72.7) |
| Other co-morbidity | 38 (14.8) |
| **Complications (multiple response)** |  |
| Hematuria | 10 (4.1) |
| Hematochezia | 2 (0.8) |
| Anaemia | 42 (17.2) |
| Hypertension | 28 (11.5) |
| Others | 14 (5.5) |
| **Traditional Chinese Medicine** |  |
| Users | 42 (16.9) |
| Non-users | 214(83.1) |
| **Time since diagnosis** |  |
| < 6 months | 31 (12.1) |
| 6-12 months | 67 (26.2) |
| 1-5 year | 95 (37.1) |
| >5 year | 63 (24.6) |
